# Attention deficit hyperactivity disorder symptoms as antecedents of later psychotic outcomes in 22q11.2 deletion syndrome

**DOI:** 10.1016/j.schres.2018.07.044

**Published:** 2019-02

**Authors:** Maria Niarchou, Samuel J.R.A. Chawner, Ania Fiksinski, Jacob A.S. Vorstman, Johanna Maeder, Maude Schneider, Stephan Eliez, Marco Armando, Maria Pontillo, Stefano Vicari, Donna M. McDonald-McGinn, Beverly S. Emanuel, Elaine H. Zackai, Carrie E. Bearden, Vandana Shashi, Stephen R. Hooper, Michael J. Owen, Raquel E. Gur, Naomi R. Wray, Marianne B.M. van den Bree, Anita Thapar, International 22q11.2 Deletion Syndrome Brain and Behavior Consortium

**Affiliations:** a Medical Research Council Centre for Neuropsychiatric Genetics and Genomics, Division of Psychological Medicine and Clinical Neurosciences, Cardiff University, Cardiff, United Kingdom; b Institute for Molecular Bioscience, University of Queensland, Brisbane, Australia; c Department of Psychiatry, Rudolf Magnus Institute of Neuroscience, University Medical Center Utrecht, Utrecht, the Netherlands; d Clinical Genetics Research Program, Centre for Addiction and Mental Health, Toronto, Ontario, Canada; e The Dalglish Family 22q Clinic for 22q11.2 Deletion Syndrome, Toronto General Hospital, University Health Network, Toronto, Ontario, Canada; f The Hospital for Sick Children, Toronto, Canada; g Department of Psychiatry, University of Geneva, Geneva, Switzerland; h Paediatric Hospital Bambino Gesu, Rome, Italy; i Division of Human Genetics, The Children's Hospital of Philadelphia, Philadelphia, PA, USA; j Semel Institute for Neuroscience and Human Behavior and Department of Psychology, UCLA, CA, USA; k Department of Pediatrics, Duke University School of Medicine, Durham, USA; l Department of Allied Health Sciences, University of North Carolina School of Medicine, Chapel Hill, NC, USA; m Department of Pediatrics, Perelman School of Medicine, University of Pennsylvania, Philadelphia, PA, USA; n Department of Psychiatry, Neuropsychiatry Section, Perelman School of Medicine, University of Pennsylvania, Philadelphia, PA, USA; o Department of Child and Adolescent Psychiatry, The Children's Hospital of Philadelphia, Philadelphia, PA, USA

**Keywords:** ADHD, 22q11.2DS, Psychotic symptoms, Inattention

## Abstract

Individuals with 22q11.2 Deletion Syndrome (22q11.2DS) are at substantially heightened risk for psychosis. Thus, prevention and early intervention strategies that target the antecedents of psychosis in this high-risk group are a clinical priority. Attention Deficit Hyperactivity Disorder (ADHD) is one the most prevalent psychiatric disorders in children with 22q11.2DS, particularly the inattentive subtype. The aim of this study was to test the hypothesis that ADHD inattention symptoms predict later psychotic symptoms and/or psychotic disorder in those with 22q11.2DS. 250 children and adolescents with 22q11.2DS without psychotic symptoms at baseline took part in a longitudinal study. Assessments were performed using well-validated structured diagnostic instruments at two time points (T1 (mean age = 11.2, SD = 3.1) and T2 (mean age = 14.3, SD = 3.6)). Inattention symptoms at T1 were associated with development of psychotic symptoms at T2 (OR:1.2, *p* = 0.01) but weak associations were found with development of psychotic disorder (OR:1.2, *p* = 0.15). ADHD diagnosis at T1 was strongly associated with development of psychotic symptoms at T2 (OR:4.5, *p* < 0.001) and psychotic disorder (OR:5.9, *p* = 0.02). Our findings that inattention symptoms and the diagnosis of ADHD are associated with subsequent psychotic outcomes in 22q11.2DS have important clinical implications. Future studies examining the effects of stimulant and other ADHD treatments on individuals with 22q11.2DS are warranted.

## 1 Introduction

22q11.2 Deletion Syndrome (22q11.2DS) is diagnosed typically on the basis of its clinical presentation together with laboratory evidence of a deletion or Copy Number Variant (CNV) at band q11.2 on chromosome 22. Approximately 1 in 4000 individuals are affected by 22q11.2DS, rendering this the most common chromosomal microdeletion syndrome (McDonald-McGinn et al., 2015). While the microdeletions range in size from 0.7 to 3 million base (MB) pairs, the majority of patients (~85%) have a 3 MB deletion (Guna et al., 2015). The physical, cognitive and psychiatric manifestations associated with 22q11.2DS are variable and involve multiple systems including immune, cardiac, palatal, gastrointestinal and endocrine deficits, regardless of deletion size (Shprintzen, 2008). An association between the 22q11.2 deletion and psychosis has long been recognised (Rees et al., 2014). Approximately 1 in 4 individuals with 22q11.2DS develop schizophrenia (e.g. (Monks et al., 2014)) and around 1 in 100 individuals with schizophrenia have been found to carry the 22q11.2 deletion (Costain et al., 2013). This means that those with 22q11.2DS are at substantially elevated risk of developing schizophrenia spectrum disorders with an onset typically occurring after mid-late adolescence.

Numerous studies of individuals with 22q11.2DS have observed a range of psychiatric and cognitive problems in childhood and adolescence; that is, prior to the typical age of onset for psychosis. These include anxiety disorders, Autism Spectrum Disorder (ASD) and intellectual disability (Schneider et al., 2014). Attention Deficit Hyperactivity Disorder (ADHD) is one of the most prevalent psychiatric disorders in childhood occurring in around 40% of individuals with 22q11.2DS (Niarchou et al., 2014; Schneider et al., 2014). Although the psychosis phenotype in 22q11.2DS is largely similar to individuals without the deletion (e.g., (Bassett et al., 2005)), the ADHD phenotype differs. Those with 22q11.2DS show more pronounced inattention symptoms than individuals with ADHD from clinically ascertained and general population samples (e.g., (Niarchou et al., 2015)). Attentional impairments are a central characteristic of schizophrenia and ADHD. Also, inattention symptoms have been shown to be antecedents of psychosis in studies of childhood-onset schizophrenia (e.g., (Alaghband-Rad et al., 1995)) as well as in studies of individuals with prodromal clinical psychosis (Pukrop et al., 2007) and of those with subclinical psychotic symptoms (e.g., (Niarchou et al., 2013)). Prior cross-sectional investigation also suggests that ADHD inattention symptoms are associated with subthreshold psychosis in 22q11.2DS (Niarchou et al., 2017a). To address the hypothesis that ADHD is an antecedent of psychosis in children and adolescents with 22q11.2 DS, we employed the first multisite and largest longitudinal study of 22q11.2DS to date to investigate this question. Here, in a sample that was first assessed at age 18 years or younger, we investigate whether childhood ADHD diagnosis and inattention symptoms are early indicators of psychosis. We also assess whether changes in inattention symptom levels and ADHD diagnosis are associated with later psychosis.

## 2 Method

### 2.1 22q11.2DS sample

The International 22q11.2 Deletion Syndrome Brain Behavior Consortium (IBBC) was established in 2013 with the aim of harmonizing existing well-characterized cohorts of participants with 22q11.2DS with both phenotypic and genotypic data available (Gur et al., 2017). For the current study, participants were recruited from 6 IBBC sites (Table 1). All participants had 22q11.2 microdeletion that was confirmed via the IBBC quality control procedures (i.e., whole genome sequencing and/or Affymetrix 6.0 microarrays as well as with available multiplex ligation dependent probe amplification (MLPA) and heat-map data from microarrays) (Gur et al., 2017). Participants were included if they underwent a comprehensive structured psychiatric assessment using a validated instrument that would provide information on ADHD diagnosis/symptoms and psychotic symptoms, if longitudinal data were available (i.e., at least one time point follow up of ADHD symptoms and psychotic symptoms/psychotic disorder) and if their age at the first-time point was ≤18 years old. This study focuses on the development of psychotic symptoms; therefore, to adopt a clearer design, those who reported any subclinical psychotic symptoms at T1 (*n* = 73) were excluded from the main analyses but included in sensitivity analyses (see statistical analyses section below).

**Table 1:** Measures used in the sites included in the study from the International Brain and Behavior Consortium in 22q11.2 Deletion Syndrome (IBBC).

Site	Sample size	Measures
Cardiff	53	CAPA
Durham, N.C.	48	C-DISC
Geneva	71	DICA. K-SADS psychosis supplement
Philadelphia	38	K-SADS, SCID
Rome	37	K-SADS
Utrecht	76	K-SADS

**Abbreviations**: CAPA = Child and Adolescent Psychiatric Assessment (Angold et al., 1995), K-SADS = Schedule for Affective Disorders and Schizophrenia for School-Age Children (Kaufman et al., 1997), SCID = Structured Clinical Interview for DSM-IV Axis I Disorders (First et al., 1996), DICA = Diagnostic Interview for Children and Adolescents (Reich, 2000), C-DISC = Computerized Diagnostic Interview Schedule for Children (Shaffer et al., 1993).

The study was approved by the appropriate local ethics committees and institutional review boards. Each participant and his or her caregiver, when appropriate, provided informed written consent/assent to participate prior to recruitment.

### 2.2 Psychiatric measures

Assessments were conducted using well-validated structured diagnostic instruments (Table 1). ADHD symptoms and diagnoses compatible with DSM-IV-TR diagnostic criteria were obtained using standard approaches for this age group -i.e., parent reported interviews. Psychotic symptoms were assessed using self- and parent-reports. If either participants or their parents reported psychotic symptoms, then these were counted as present. We only included the positive symptoms of psychosis in our analyses (i.e., hallucinations, delusions and thought interference). Phenomena were not coded as psychotic symptoms if they were attributed to hypnagogic and hypnopompic states, fever or substance use. Due to the different assessment methods and range of questions asked between the different sites, presence of any subclinical/clinical psychotic symptom was coded as 1 (present) vs. 0 (absent), instead of using a continuous scale. The ratings of psychotic disorders were harmonized across the sites as part of the IBBC initiative (Gur et al., 2017). Data on ADHD symptoms were also harmonized, with sites completing information on a specific list of symptoms (Table S1). A total inattention symptom score was obtained by summarizing the number of inattention symptoms that were reported as present. If at least one missing value was present, the total inattention symptom score was reported as missing, hence the different numbers reported for ADHD diagnosis and inattention symptoms. The primary predictor variables were inattention symptoms (total score) and ADHD diagnosis (present vs. absent). The outcome variables were 1. psychotic symptoms (present vs. absent) and 2. psychotic disorder (present vs. absent). Psychotic disorder included schizophrenia, schizophreniform disorder, schizoaffective disorder, psychotic disorder NOS, delusional disorder (type or unknown), and brief psychotic disorder with at-risk status as defined by the DSM-5. For the purpose of sensitivity analyses, hyperactive-impulsiveness and total ADHD symptom scores were also considered as predictor variables.

### 2.3 Confounders

Standardized IQ scores were available across the sites using age-appropriate Wechsler scales and were examined as confounders. Taking into account that intellectual disability is frequently present in 22q11.2DS, some sites assessed the presence/absence of ADHD symptoms taking into account whether the individual with 22q11.2DS had intellectual disability. To account for this, we included ‘site assessment differences’ as a covariate variable. We also included age at baseline and sex as covariates.

### 2.4 Statistical analyses

Logistic regressions were conducted to examine whether T1 inattention symptoms and ADHD diagnosis (independent variables) predicted T2 outcome (dependent variable) status (0 = individuals without psychotic symptoms, 1 = individuals with psychotic symptoms and 0 = individuals without psychotic disorder, 1 = individuals with psychotic disorder). We repeated the analyses by also including age, sex, IQ and the variable ‘site assessment differences’ (see confounders above) as covariates. We also examined whether longitudinal change in inattention symptoms and ADHD diagnosis were associated with psychotic symptoms and/or any psychotic disorder at T2. Change in inattention symptoms in relation to psychotic symptoms and psychotic disorder was examined using principal components analysis (PCA) following previously reported methods (Chawner et al., 2017; Niarchou et al., 2013). The PCA included inattention symptoms at T1 and inattention symptoms at T2. Two factors were identified from the PCA, one corresponding to the average of ADHD symptoms at T1 and T2 (f1) and the other one (f2) representing change over time. Logistic regression analyses were used to examine the associations between change over time (f2) and psychotic symptoms/psychotic spectrum disorders at T2 after adjusting for average ADHD symptoms (f1) as well as sex, IQ, age and site assessment differences. The advantage of the PCA method is that the two factors (i.e., average and change) are uncorrelated in the regression model. To examine change of ADHD diagnosis between T1 and T2 we constructed a categorical variable (presence of ADHD at neither time point = 0, ADHD at T1 but not at T2 = 1, ADHD at T2 but not T1 = 2, ADHD at both time points = 3). Logistic regression analyses were used where the categorical variable was the predictor and psychotic symptoms/psychotic disorder were the outcome variables while adjusting for sex, IQ, age and site assessment differences. Where psychotic disorder was used as an outcome, due to the small sample size of cases with psychotic disorder, the maximum likelihood estimates tended to infinity, and in this case we used Firth's method (Firth, 1993) instead (Heinze and Schemper, 2002). As a sensitivity analysis, we repeated these analyses by also examining hyperactive-impulsiveness symptoms and total ADHD symptoms. We also examined whether there were differences in inattention symptoms and ADHD diagnosis in individuals who at T1 reported psychotic symptoms compared with individuals that did not report psychotic symptoms at T1.

## 3 Results

### 3.1 Sample description

The sample started with a total of 323 individuals (49% males) aged 11.8 years at the first assessment (SD = 3.3) and 15.1 years (SD = 4.0) at the second assessment. Excluding 73 participants who reported psychotic symptoms at T1, our final sample included 250 individuals (49% males) with complete data on psychotic symptoms and ADHD diagnosis and 188 individuals (47% males) with complete data on psychotic symptoms and inattention symptoms (Table 2). The mean age at assessment for individuals at T1 was 11.2 years (SD = 3.1, age range 6 to 18) and at T2 was 14.3 years (SD = 3.6, age range 6 to 18). The mean follow-up time across sites was 3.19 years (SD = 1.5). Of those with psychotic symptoms at T2, 71% also had an ADHD diagnosis at T2 and of those with psychotic disorder at T2, 63% also had an ADHD diagnosis (Table 2).

**Table 2:** Sample size and descriptive statistics of individuals with 22q11.2DS included in the study.

Time point 1 (T1)	Time point 2							
		Age –T1	Age – T2	Sex	PS (*N* = 45)	No PS (*N* = 143)	PSD(N = 14)	No PSD (*N* = 174)
	Ν	Mean(SD)	Mean(SD)	% males	Mean(SD)	Mean(SD)	Mean(SD)	Mean(SD)
ADHD inattention symptoms	188	11.5(3.2)	14.6(3.8)	47%	5.4(2.4)	3.5(3.0)	4.9(2.2)	3.9(3.0)


	N	Mean(SD)	Mean(SD)	% males	PS (*N* = 51)	No PS (*N* = 199)	PSD(*N* = 16)	No PSD (*N* = 234)
					%	%	%	%
ADHD diagnosis	250	11.2(3.1)	14.3(3.6)	49%	71%	35%	63%	41%

**Notes**: N does not include individuals with psychotic symptoms at time point 1. N of individuals with data on inattention symptoms is smaller because not all had information on inattention symptoms.

**Abbreviations**: PS = psychotic symptoms (present/absent), PSD = psychotic disorder (present/absent), % = percentage of those with PS/No PS/PSD/No PSD and an ADHD diagnosis.

### 3.2 Associations between inattention symptoms and ADHD at T1 and development of psychosis at T2

ADHD inattention symptoms and ADHD diagnosis at T1 were associated with development of psychotic symptoms at T2 (OR = 1.22, *p* = 0.01 and OR = 4.5, *p* < 0.001 respectively) (Table 3). There was no evidence for associations between inattention symptoms at T1 and psychotic disorder at T2 (OR = 1.18, *p* = 0.15). ADHD diagnosis at T1 was associated with psychotic disorder at T2 (OR = 5.92, *p* = 0.02) (Table 3).

**Table 3:** ADHD symptoms/diagnosis at T1 before and after adjusting for confounders in relation to psychotic symptoms/psychotic disorder at T2.

Time point 1	Psychotic symptoms								
	Time point 2								
	Unadjusted			Adjusted^a^			Adjusted^b^		
	Odds ratio	95%CI	p	Odds ratio	95%CI	p	Odds ratio	95%CI	p
ADHD inattention symptoms	1.26	1.11–1.43	<0.001	1.29	1.13–1.48	<0.001	1.22	1.06–1.41	0.01
ADHD diagnosis	4.42	2.31–8.85	<0.001	6.05	2.93–13.25	<0.001	4.45	2.09–10.08	<0.001


Time point 1	Psychotic disorder								
	Time point 2								
	Unadjusted			Adjusted^a^			Adjusted^b^		
	Odds ratio	95%CI	p	Odds ratio	95%CI	p	Odds ratio	95%CI	p
ADHD inattention symptoms	1.12	0.93–1.36	0.24	1.16	0.94–1.44	0.17	1.18	0.94–1.48	0.15
ADHD diagnosis	2.40	0.86–7.25	0.10	3.61	1.21–11.8	0.03	5.92	1.52–28.56	0.02

Notes: N does not include individuals with psychotic experiences at time point 1. ADHD inattention symptoms and ADHD diagnosis are in separate logistic models. Abbreviations: ADHD = Attention Deficit Hyperactivity Disorder, ^a^ age, sex and IQ included as covariates, ^b^ age, sex, IQ and assessment differences included as covariates.

Sensitivity analyses revealed no association between T1 hyperactive-impulsiveness symptoms at T1 and psychotic symptoms at T2 (OR = 1.15, *p* = 0.27) or with psychotic disorder at T2 (OR = 1.17, *p* = 0.33) (Table S2). There was weak evidence for associations between total ADHD symptoms (i.e. hyperactive-impulsiveness and inattention symptom scores combined) and development of T2 psychotic symptoms (OR = 1.11, *p* = 0.07) but no evidence for associations with psychotic disorder at T2 (1.10, *p* = 0.26) (Table S2).

As a further sensitivity analysis, we also compared individuals with and without psychotic symptoms at T1 (Table S3). We found significant age and IQ differences, with the group reporting psychotic symptoms at T1 being older than the group without psychotic symptoms at T1 and of lower mean IQ score. There were no significant mean differences in terms of inattention symptoms and ADHD diagnosis.

Table S4 shows the summary statistics for change in inattention symptom levels and ADHD diagnosis over time in relation to psychotic symptoms at T2. There was no evidence that change over time in inattention symptom levels or ADHD diagnosis was associated with psychotic symptoms or psychotic disorder at T2 (Table 4). Results were the same for our sensitivity analyses with hyperactive-impulsive and total ADHD symptoms (Table S5).

**Table 4:** Relationship between mean levels and change over time in ADHD symptoms/diagnosis and psychotic symptoms/psychotic disorder at T2.

Predictor variables	Outcome variable: psychotic symptoms at time point 2			
	Average (f1)		Change (f2)	
	Odds ratio (95%CI)	*p*-Value	Odds ratio (95%CI)	*p*-Value
ADHD inattention symptoms	1.51(1.05–2.22)	0.03	1.14(0.48–2.73)	0.77
	Change			
	Odds ratio (95%CI)		*p*-Value	
ADHD diagnosis- Categories				
ADHD at T1 but not T2	3.52(0.84–14.49)		0.08	
ADHD at T2 but not T1	5.74(0.64–40.0)		0.08	
ADHD at both time points	9.79(3.59–30.52)		<0.001	


	Outcome variable: psychotic disorder at time point 2			
	Average (f1)		Change (f2)	
	Odds ratio (95%CI)	*p*-Value	Odds ratio (95%CI)	*p*-Value
ADHD inattention symptoms	1.31(0.72–2.41)	0.37	1.61(0.37–7.41)	0.53
	Change			
	Odds ratio (95%CI)		*p*-Value	
ADHD diagnosis- Categories				
ADHD at T1 but not T2	0.65(0.00–6.69)		0.77	
ADHD at T2 but not T1	1.68(0.01–21.13)		0.76	
ADHD at both time points	4.76(1.37–18.91)		0.01	

**Notes**: N does not include individuals with psychotic symptoms at time point 1, f1 = factor 1 identified from the Principal Components Analysis (PCA) and captures mean levels across T1 and T2, f2 = factor 2 identified from the PCA and captures changes between T1 and T2.

## 4 Discussion

In the largest longitudinal study examining the presence of ADHD symptoms and diagnosis in individuals with 22q11.2DS to date, we observed that inattention symptom levels and ADHD diagnosis predicted the development of psychotic symptoms and were weakly associated with psychotic disorder. Moreover, we found that the presence of inattention symptoms at any time point rather than the change in inattention symptom levels over time was associated with psychotic symptoms. The evidence was weaker for the outcome of psychotic disorder, but this is likely due to low power, since in this young sample (mean age 14.3 years) only 6% were diagnosed with psychotic disorder.

These longitudinal findings are in accordance with our previous study that found cross-sectional associations between inattention symptoms and psychotic symptoms in individuals with 22q11.2DS (Niarchou et al., 2017a). Previous population-based (e.g., (Niarchou et al., 2013)) and clinical studies (Pukrop et al., 2007) also have observed that childhood inattention symptoms are an antecedent to psychosis.

There are a number of potential explanations for our findings. One is that the 22q11.2 deletion increases risk for inattention symptoms and ADHD, which in turn increase risk for psychotic outcomes (Marsh and Williams, 2006). Another is that inattention symptoms or an ADHD diagnosis in the context of 22q11.2DS are prodromal or premorbid forms of schizophrenia rather than ADHD per se. For instance, Fletcher and Frith (2009) suggested that psychotic symptoms are the result of an abnormal formation of beliefs about the world (i.e., false prediction errors). In this way, individuals with inattention symptoms might direct their attention to less relevant or too many environmental cues and in turn might perceive and interpret environmental stimuli as more unusual and salient, which would predispose them to having psychotic symptoms. Therefore, these inattention symptoms might be indicators of abnormal probabilistic learning that has been observed in schizophrenia (e.g., (Reddy et al., 2016)). However, we cannot exclude the possibility that the associations between inattention symptoms and psychotic outcomes also reflect shared genetic variance, especially given evidence for genetic overlap between ADHD and schizophrenia (Demontis et al., 2017; Hamshere et al., 2013).

On the other hand, we did not observe cross-sectional associations between inattention symptoms/ADHD diagnosis and psychotic symptoms at T1. ADHD diagnosis was equally comorbid in those with and without psychotic symptoms and the mean levels of inattention symptoms were similar between the two groups. A previous study that examined the dimensional structure of a wide spectrum of psychopathology in 22q11.2DS has found evidence of a general psychopathology factor in addition to more specific factors (i.e., anxiety, mood, ADHD and psychosis) (Niarchou et al., 2017b). Therefore, one potential explanation of our findings is that at an earlier age, ADHD symptoms and psychotic symptoms also indicate the individual's general propensity for psychopathology and as individuals approach the age of onset for risk of psychosis, certain symptoms become more specific to psychosis. Finally, taking into account that psychiatric conditions can co-occur, it may not be ADHD per se, or any other psychiatric disorder, but rather the severity of presentation and/or the cumulative contributions of increasing psychiatric conditions/severity that increases risk for psychotic outcomes.

We did not observe association between hyperactive-impulsive symptoms and psychotic outcomes, which accords with cross-sectional studies of 22q11.2DS (Niarchou et al., 2017a) and population-based studies (Hurtig et al., 2011). As has been previously suggested (Niarchou et al., 2017a), this could be due to differences in the way dopaminergic function acts between schizophrenia and ADHD, with dopamine hypo-activity being more likely linked to the hyperactivity-impulsiveness aspects of ADHD (Dichter et al., 2012) and dopamine hyperactivity to schizophrenia (Howes and Kapur, 2009).

### 4.1 Clinical implications

Our study is the first to show that inattention symptom levels and ADHD diagnosis in those with 22q11.2DS are associated with later emerging psychotic outcomes. If inattention and ADHD are risk factors for future psychosis, then effective treatment is a priority for reducing the risk of psychosis in this high-risk group. However, if ADHD is a prodromal feature of psychosis in this group, then taking into account that stimulant medication is often prescribed for ADHD, future studies are needed to examine potential effects of such treatment in individuals with 22q11.2DS. A randomized controlled trial in thirty-four children with 22q11.2DS and ADHD indicated that methylphenidate can be safe and effective after a 6-month treatment and led to a 40% reduction of ADHD symptoms as reported by parents (Green et al., 2011). Interestingly, all subjects had at least one side effect and approximately 40% exhibited depressive-like symptoms after treatment. Although informative, the sample size as well as the follow-up time were limited in this study. Further studies are warranted to examine the effect of stimulants and other ADHD-treatments in 22q11.2DS.

Although the link between ADHD and psychosis is not adequately studied (Pallanti and Salerno, 2015), our findings support those from previous patient registry and high-risk studies in populations without the 22q11.2 deletion that have observed associations between ADHD, early attentional impairments and later psychosis (Dalsgaard et al., 2014; Erlenmeyer-Kimling et al., 2000), as well as comorbidity between ADHD and psychosis (Levy et al., 2015). Taking into account that 22q11.2DS is a rare, large effect size mutation that serves as a powerful model for examining early antecedents of psychosis, our findings further point to the possibility that some patients with ADHD might present with psychotic symptoms at follow-up. The findings also highlight the need for further studies in order to better understand the relationship between ADHD and psychosis.

### 4.2 Limitations

Although this study benefitted from recruitment of a number of sites of the 22q11.2 IBBC, resulting in a relatively large sample, the study may have been underpowered for some analyses (e.g., psychotic disorder). Moreover, the mean age at follow up was 14.3 years and therefore the individuals with 22q11.2DS had not yet passed the peak age of onset for schizophrenia. Therefore, the associations that we report are likely to represent an underestimate. Another limitation is that we could not consider the impact of medication, since at the time of this analysis medication information was not consistently reported from all sites. However, failing to adjust for medication is more likely to have attenuated the magnitude of association between ADHD symptoms and psychotic outcomes. Although the assessments were conducted by experienced clinicians and psychologists, we cannot exclude the possibility of diverse diagnostic practices across sites that might have influenced our findings. However, we attempted to account for differences between centres in our analyses and did not find that site assessment differences explained our findings. Ascertainment bias is also likely, considering that genetic testing was conducted on the basis of a phenotype that was sufficient to warrant genetic testing (e.g., heart defects, developmental delay). Finally, taking into account that comorbidity is common in 22q11.2DS, it could be that other disorders (e.g., intellectual disability, autism), in addition to ADHD, might be longitudinally associated with psychotic outcomes in 22q11.2DS. However, this question was outside the remits of this study. Also, it could be argued that ADHD is more easily amenable to symptom reduction by treatment than other potential clinical risk factors for psychosis in 22q11.2DS (e.g. intellectual disability). Interestingly, a recent study on 89 children with 22q11.2DS did not find longitudinal associations between autism spectrum disorders and psychosis (Fiksinski et al., 2017).

### 4.3 Conclusions

Our study is the first to examine the longitudinal associations between ADHD symptoms and psychotic outcomes in 22q11.2DS. Our findings that inattention symptoms and ADHD diagnosis were associated with subsequent psychotic symptoms and psychotic disorder in 22q11.2DS have important clinical implications. Future studies examining the effects of ADHD medication in individuals with 22q11.2DS are warranted.

## Author contributions

Study concept and design: Maria Niarchou, Anita Thapar.

Acquisition of data: Maria Niarchou, Marianne B.M. van den Bree, Samuel J.R.A. Chawner, Ania M. Fiksinski, Jacob A.S. Vorstman, Maude Schneider, Stephan Eliez, Marco Armando, Maria Pontillo, Donna M. McDonald-Mcginn, Beverly S. Emanuel, Elaine H. Zackai, Carrie Bearden, Vandana Shashi, Stephen Hooper, Michael J. Owen, Raquel E. Gur.

Analysis of data: Maria Niarchou, Naomi Wray, Marianne B.M. van den Bree, Anita Thapar.

Interpretation of data: Maria Niarchou, Naomi Wray, Marianne B.M. van den Bree, Anita Thapar, Michael J. Owen, Raquel E. Gur.

Critical revision of the manuscript for important intellectual content: All authors.

## Funding

This study was funded by the National Institute of Mental Health grants USA U01MH101719, U01MH0101720, Wellcome Trust Fellowship (M.N. 110222/Z/15/Z), R01 MH085953 (CEB), U54 EB020403 (CEB), Swiss National Science Foundation (324730_144260) to SE, National Center of Competence in Research (NCCR) “Synapsy-The Synaptic Bases of Mental Diseases” (51NF40-158776) to S.E.

## Funding sources

The funding sources had no participation in any of the aspects of this study (e.g., data collection, analysis etc.).

## Conflict of interest disclosures

None reported.

## Appendix A Supplementary data

Supplementary tables Image 1
