# Supplementary material for: Attention deficit hyperactivity disorder symptoms as antecedents of later psychotic outcomes in 22q11.2 deletion syndrome
Source: Schizophr Res. 2019 Feb;204:320–5. doi: 10.1016/j.schres.2018.07.044 (PMC6406019; doi:10.1016/j.schres.2018.07.044)
Supplement: Supplementary file 1 — Supplementary tables [file mmc1.docx]

Supplementary material

**Table S1**. List of ADHD symptoms assessed

| **List of ADHD symptoms** |
| --- |
| **Inattention symptoms** |
| Frequent trouble paying attention to school/work/chores |
| Frequent trouble paying attention to enjoyable activities |
| Difficulty paying attention to instructions |
| Difficulty sustaining attention to activities requiring mental effort |
| Often losing things/ making careless mistakes |
| Difficulty planning/organizing |
| Daydreaming/trouble listening |
| Often forgetting |
| Often distracted |
| **Hyperactivity-impulsivity symptoms** |
| Difficulty remaining still |
| Fidgety |
| Always on the go |
| Climbing on things/ running around when it’s not appropriate |
| Difficulty with quiet activities |
| Extremely talkative |
| Blurting out answers/interrupting people when they are talking |
| Trouble waiting for turn |

**Table S2. Hyperactivity-impulsiveness and total ADHD symptoms at T1 before and after adjusting for confounders in relation to psychotic symptoms/ any psychotic disorder at T2.**

|  | **Psychotic symptoms** | | | | | | | | |
| --- | --- | --- | --- | --- | --- | --- | --- | --- | --- |
|  | **Time point 2** | | | | | | | | |
| **Time point 1** | **Unadjusted** | | | **Adjusted^a^** | | | **Adjusted^b^** | | |
|  | **Odds ratio** | **95%CI** | **p** | **Odds ratio** | **95%CI** | **p** | **Odds ratio** | **95%CI** | **p** |
| **ADHD hyperactivity- impulsiveness symptoms** | 0.96 | 0.78-1.16 | 0.67 | 0.96 | 0.77-1.18 | 0.72 | 1.15 | 0.90-1.47 | 0.27 |
| **ADHD total symptoms** | **1.10** | **1.01-1.20** | **0.03** | **1.12** | **1.02-1.24** | **0.02** | 1.11 | 0.99-1.24 | 0.07 |
|  | **Any psychotic disorder** | | | | | | | | |
|  | **Time point 2** | | | | | | | | |
| **Time point 1** | **Unadjusted** | | | **Adjusted^a^** | | | **Adjusted^b^** | | |
|  | **Odds ratio** | **95%CI** | **p** | **Odds ratio** | **95%CI** | **p** | **Odds ratio** | **95%CI** | **p** |
| **ADHD hyperactivity-impulsiveness symptoms** | 1.03 | 0.76-1.32 | 0.82 | 1.09 | 0.78-1.46 | 0.58 | 1.17 | 0.83-1.61 | 0.33 |
| **ADHD total symptoms** | 1.05 | 0.92-1.19 | 0.47 | 1.10 | 0.94-1.29 | 0.22 | 1.10 | 0.93-1.29 | 0.26 |
| Notes: N does not include individuals with psychotic experiences at time point 1. Abbreviations: ADHD=Attention Deficit Hyperactivity Disorder, ^a^ age, sex and IQ included as covariates, ^b^ age, sex, IQ and assessment differences included as covariates. In bold, associations that passed the significance threshold p<0.05. | | | | | | | | | |

**Table S3. Sensitivity analysis – comparing individuals with and without psychotic symptoms at T1.**

|  | **Time point 1** | |  |  |
| --- | --- | --- | --- | --- |
|  | **PS (N=73)** | **No PS (N=250)** |  |  |
|  | **N(%)** | **N(%)** | **χ^2^, z** | **p** |
| **Sex(males -%)** | 48% | 49% | 0.04 | 0.85 |
|  | **Mean(SD)** | **Mean(SD)** | **χ^2^, z** | **p** |
| **Age** | **14.0(2.6)** | **11.2(3.1)** | **43.3** | **<0.001** |
| **IQ** | **69.8(14.3)** | **74.4(14.0)** | **5.50** | **0.02** |
| **ADHD inattention symptoms** | 3.51(2.9) | 4.0(3.0) | 1.13 | 0.29 |
|  | **%*** | **%*** |  |  |
| **ADHD diagnosis** | 34% | 42% | 1.56 | 0.21 |
| **Abbreviations:** PS=psychotic symptoms  **Notes:** *: percentage of those with/without psychotic symptoms who also had an ADHD diagnosis. | | | | |

**Table S4.** Summary statistics of change in inattention symptoms and ADHD diagnosis over time in relation to psychotic experiences.

|  |  | **Psychotic symptoms at time point 2** | | **No psychotic symptoms at time point 2** | |
| --- | --- | --- | --- | --- | --- |
|  |  | **N** | **Mean(SD)** | **N** | **Mean(SD)** |
| **ADHD inattention symptoms** | **T1** | 34 | 5.0(2.5) | 122 | 3.4(2.9) |
|  | **T2** |  | 4.9(2.6) |  | 3.1(3.0) |
|  |  |  | **%** |  | **%** |
| **ADHD diagnosis** | **T1** | 40 | 32% | 177 | 68% |
|  | **T2** |  | 35% |  | 65% |
|  |  | **Any psychotic disorder** | | **No psychotic disorder** | |
|  |  | **N** | **Mean(SD)** | **N** | **Mean(SD)** |
| **ADHD inattention symptoms** | **T1** | 9 | 4.1(2.3) | 147 | 3.7(3.0) |
|  | **T2** |  | 4.0(3.1) |  | 3.4(3.0) |
|  |  |  | **%** |  | **%** |
| **ADHD diagnosis** | **T1** | 9 | 9% |  | 91% |
|  | **T2** |  | 11% |  | 89% |
| **Notes:** N does not include individuals with psychotic symptoms at time point 1. Different sample sizes than Table 2 because not everyone had an inattention symptom count score at T2. N is number of individuals with/without psychotic symptoms/psychosis spectrum disorder. %: % of those with/without psychotic symptoms/psychosis spectrum disorder out of those with an ADHD diagnosis. N=number of individuals with/without psychotic symptoms/psychotic disorder. | | | | | |

**Table S5.** Relationship between mean levels and change over time in hyperactivity-impulsiveness and total ADHD symptoms and psychotic symptoms/ any psychosis spectrum disorder at T2.

|  | **Psychotic symptoms at Time point 2** | | | | |
| --- | --- | --- | --- | --- | --- |
|  | **Average (f1)** | | | **Change (f2)** | |
|  | **Odds ratio (95%CI)** | **p-value** | | **Odds ratio (95%CI)** | **p-value** |
| **ADHD hyperactivity-impulsiveness symptoms** | 1.21(0.75-1.90) | 0.42 | | 1.03(0.48-2.20) | 0.94 |
| **ADHD total symptoms** | 1.25(0.79-1.96) | 0.33 | | 1.12(0.41-3.11) | 0.83 |
|  | **Any psychosis spectrum disorder at Time point 2** | | | | |
|  | **Average (f1)** | | **Change (f2)** | | |
|  | **Odds ratio (95%CI)** | **p-value** | **Odds ratio (95%CI)** | | **p-value** |
| **ADHD hyperactivity-impulsiveness symptoms** | 1.36(0.74-2.37) | 0.28 | 0.62(0.21-1.85) | | 0.39 |
| **ADHD total symptoms** | 1.28(0.66-2.38) | 0.44 | 2.07(0.43-11.25) | | 0.38 |
| **Notes:** N does not include individuals with psychotic symptoms at time point 1, f1=factor 1 identified from the Principal Components Analysis (PCA) and captures mean levels across T1 and T2, f2=factor 2 identified from the PCA and captures changes between T1 and T2. In bold are values p<0.05. | | | | | |
